# Clinical implementation of a multidisciplinary pipeline for genome sequencing in rare diseases: A prospective, multicenter, observational cohort study

**DOI:** 10.1002/ctm2.70401

**Published:** 2025-07-10

**Authors:** Soojin Hwang, Go Hun Seo, In Hee Choi, Seung‐Woo Ryue, Ji Young Oh, Yoo‐Mi Kim, Baik‐Lin Eun, Jung Hye Byeon, Eugu Kang, Myungshin Kim, Hoon Seok Kim, Soyoung Lee, Han Wool Kim, Dohyung Kim, Rin Khang, Jihye Kim, Dongseok Moon, Seokhui Jang, Yongjun Song, Gu‐Hwan Kim, Kyoung Bo Kim, Jun Hong Park, Seo Yeon Yang, Yoo Kyoung Choi, Su Min Ji, Oc‐Hee Kim, Mi‐Hyun Park, Hyun‐Young Park, Beom Hee Lee

**Affiliations:** ^1^ Department of Paediatrics Medical Genetics Centre, Asan Medical Centre, University of Ulsan College of Medicine Seoul South Korea; ^2^ Medical Genetics Division 3billion Inc. Seoul South Korea; ^3^ Department of Genetic Counselling University of Ulsan College of Medicine Seoul South Korea; ^4^ Department of Paediatrics Severance Children's Hospital, Yonsei University College of Medicine Seoul South Korea; ^5^ Department of Paediatrics Chungnam National University Sejong Hospital Sejong South Korea; ^6^ Department of Paediatrics Korea University College of Medicine Seoul South Korea; ^7^ Department of Laboratory Medicine Seoul St. Mary's Hospital, College of Medicine, The Catholic University of Korea Seoul South Korea; ^8^ Department of Paediatrics Hallym University Sacred Heart Hospital Anyang South Korea; ^9^ Medical Genetics Centre, Asan Medical Centre, University of Ulsan College of Medicine Seoul South Korea; ^10^ Department of Laboratory Medicine Keimyung University School of Medicine Daegu South Korea; ^11^ Department of Laboratory Medicine Jeonbuk National University Medical School and Hospital Jeonju South Korea; ^12^ Department of Precision Medicine Division of Genome Science, National Institute of Health Cheongju South Korea; ^13^ National Institute of Health Cheongju South Korea

1

Dear Editor,

Patients with rare diseases (RDs) continue to experience diagnostic delays, limited treatment options, and restricted access to personalised genetic counselling.^1^, ^2^ Genome sequencing (GS) is an effective diagnostic tool, with reported yields of up to 70%, particularly for detecting variants in non‐coding regions and complex structural variants (SVs), offering advantages over exome sequencing (ES) and chromosomal microarray (CMA).^3^ This study aimed through GS to advance precision medicine for RDs in real‐world clinical practice as a collaborative endeavour involving medical staff, geneticists, technicians and genetic counselors. From August to November 2023, a prospective, observational and multicenter study was conducted for RDs. This cohort comprised 901 participants (387 probands and 514 family members; Figure 1 and detailed methods in Supporting Information 1). The principal study outcome was the diagnostic yield from GS, with additional outcomes including secondary findings, clinical management and genetic counseling.

**FIGURE 1:**
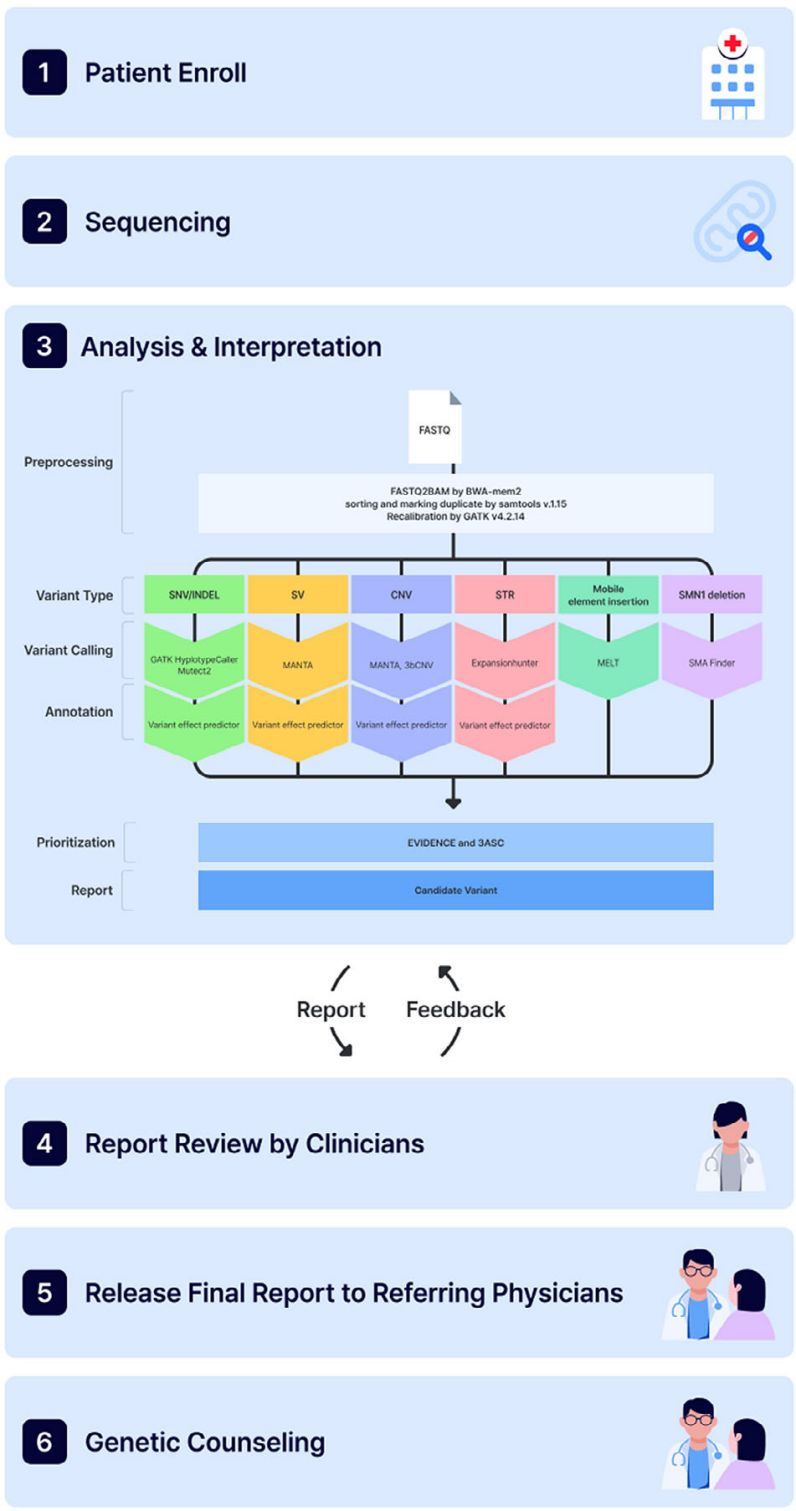
Overall flow chart for diagnosing rare diseases through genome sequencing. SNV, single‐nucleotide variant; INDEL, insertion/deletion variant; SV, structural variant; CNV, copy number variant; STR, short tandem repeat.

The 387 families included 114 single probands, 39 family duos, 217 family trios, 10 family quads and seven others (Table 1). The study population consisted of 387 probands across various age groups. Among them, seven (1.8%) were neonates, 12 (3.1%) were infants, and 175 (45.2%) were children. Thirty‐five (9.0%) were adolescents, while adults accounted for 158 (40.8%) of the study population. The median age of the 387 probands was 12.2 years (range 0 to 57). Genetic testing prior to GS was noted in 126 (32.6%) probands. The disease categories among the probands included neurodevelopmental disorders (NDD, 25.3%), ophthalmological disorders (13.7%), dysmorphic and congenital abnormalities (11.1%), neurologic disorders (10.6%), tumour syndrome (8.3%), skeletal disorders (7%), cardiovascular disorders (5.4%) and urinary tract disorders (3.9%). The average turnaround time was 53.9 ± 30.1 days from sample receipt to report delivery to the referring physicians.

**TABLE 1:** Demographic characteristics of the 387 patients with rare diseases included in this study.

Characteristic	No. of 387 probands (%)
***Sex (male:female)***	205 (52.9):182 (47.0)
***Family type***	
Proband only	114 (29.5)
Duos	39 (10.1)
Trios	217 (56.1)
Quads	10 (2.6)
Others	7 (1.8)
***Median age at sample accession: years (range)***	12.2 (0.0–57)
***Age at sample accession***	
Neonatal (from birth to 1 month)	7 (1.8)
Infancy (from more than 1 month to 1 year)	12 (3.1)
Childhood (from more than 1 year to 12 years)	175 (45.2)
Adolescent (from more than 12 years to 18 years)	35 (9.0)
Adult (from more than 18 years)	158 (40.8)
***Total number of patients with previous genetic testing***	126 (32.6)
Single gene test	32 (25.3)
Panel test	29 (23.0)
Exome sequencing test	24 (19.0)
Multiplex ligation‐dependent probe amplification	10 (7.9)
Chromosomal microarray	47 (37.3)
Karyotyping	33 (26.2)
Others	15 (11.9)
***Symptom category***	
Respiratory disorders	3 (0.8)
Neurologic disorders	41 (10.6)
Renal and urinary tract disorders	15 (3.9)
Skeletal disorders	27 (7)
Dysmorphic and congenital abnormality syndromes	43 (11.1)
Endocrine disorders	6 (1.6)
Ophthalmological disorders	53 (13.7)
Cardiovascular disorders	21 (5.4)
Gastroenterology and hepatology	6 (1.6)
Haematological and immunological disorders	9 (2.3)
Hearing and ear disorders	2 (0.5)
Neurodevelopmental disorders	98 (25.3)
Tumour syndrome	32 (8.3)
Metabolic disorders	8 (2.1)
Dermatological disorders	11 (2.8)
Growth disorders	6 (1.6)
Autoimmune or Rheumatological disorders	6 (1.6)

Diagnostic or inconclusive results (as defined in the methods section of Supporting Information 1) were reported in 27% (104/387, 95% confidence interval [CI]: 22.5%–31.3%) and 9.0% (35/387, 95% CI: 6.2%–1.2%) of the probands, respectively (Figure 2A; Tables S1 and S2 in Supporting Information 2). Of the 104 patients with diagnostic results, 80 cases (77.9%) had single‐nucleotide variants (SNVs) and small insertion/deletion variants (INDELs) in the nuclear genome, and two (1.6%) had an SNV in the mitochondrial genome. SVs were identified in 22 cases (4.9%): 14 with copy number variants (CNVs), two with complex SVs, four with repeat expansions, one with a mobile element insertion (MEI) and one with a chromosomal rearrangement. Forty‐eight variants (40.7%) were novel, and 44 (37.3%) were confirmed to be *de novo*. Sixteen out of 139 families (11.5%) with diagnostic and inconclusive results could only have been identified by GS including four deep intronic variants, a single3′ untranslated region (UTR) variant, two mitochondrial SNVs, 3 CNVs spanning less than three consecutive exons and of < 50 kb in size, four repeat expansions and two complex SVs (Table S3 in Supporting Information 2). Overall, 86 unique genetic disorders were identified in 104 patients with RDs who received diagnostic results, including one patient diagnosed with two distinct conditions, osteogenesis imperfecta type I and familial cylindromatosis. The observed inheritance patterns included 64 autosomal dominant cases (74.4%), 11 autosomal recessive (12.8%), eight X‐linked (9.3%), one digenic recessive (1.2%), and one mitochondrial (1.2%).

**FIGURE 2:**
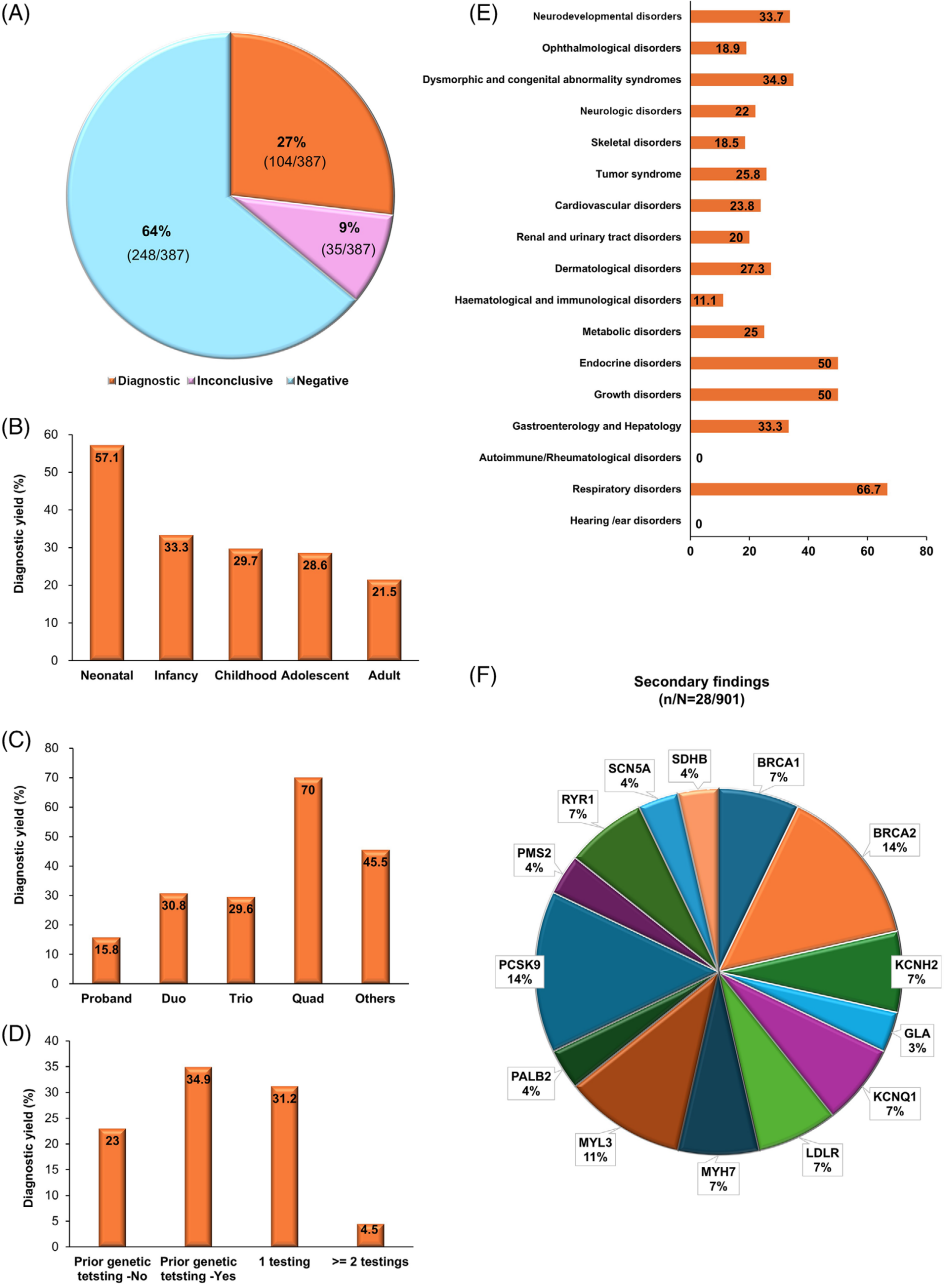
Diagnostic yield for rare diseases and secondary findings from genome sequencing. (A) Overall diagnostic yield. (B) Diagnostic yield by age group. (C) Diagnostic yield by family analysis type: proband‐only, duo (proband and one parent), trio (proband and both parents), quad (proband, both parents and one sibling) and others (proband with siblings or grandparents). (D) Diagnostic yield based on previous genetic testing. (E) Diagnostic yield by disease type. (F) Diagnostic yield for secondary findings.

Pediatric patients had a higher diagnostic yield [30.6% (70/229, 95% CI: 24.6%–36.6%)] than adults [21.5% (34/158, 95% CI: 15.1%–27.9%), *p *= .048] (Figure 2B). Single proband samples had the lowest yield of 15.8% (18/114, 95% CI: 9.1%–22.5%), while family duos and trios had yields of 30.8% (12/39, 95% CI: 16.3%–45.3%, *p *= .041) and 29.5% (64/217, 95% CI: 23.5%–35.7%, *p *= .005), respectively. Family quads had the highest yield at 70% (7/10, 95% CI: 41.6%–98.4%, *p *< .001) (Figure 2C). Patients with prior genetic testing had a higher diagnostic yield [34.9% (44/126, 95% CI: 27%–43%), *p *= .013], than those without prior testing [20.3% (60/261, 95% CI: 18%–28%)] (Figure 2D). Diagnostic yields also varied by disease category, ranging from 0% (0/6) for autoimmune/rheumatologic disorders and hearing/ear disorders to 66.7% (15/43, 95% CI: 20.7%–49.1%) for respiratory disorders (Figure 2E). In the NDD and dysmorphic and congenital abnormality syndromes that were evident mostly in pediatric patients, the diagnostic yields were high at 33.7% (33/98, 95% CI: 24.3%–43.1%) and 34.9% (15/43, 95% CI: 20.7%–49.1%), respectively. The cardiovascular and ophthalmologic disorder groups that included mainly adult patients had low diagnostic yields of 23.8% (5/21, 95% CI: 5.6%–42%) and 18.9% (10/53, 95% CI: 8.4%–29.4%), respectively.

It was noteworthy that most of the study participants (467/493 who responded to the survey,^4^, ^5^ 94.7%) expressed a desire to be informed of secondary findings based on the ACMG SF v3.2 list^6^ (Table S4 in Supporting Information 2). With an increasing probability of genetic transmission to offspring, 79.5% favoured disclosure and even for uncertain results, 73.4% wanted to be informed of the results or to join the decision‐making process. These findings suggest a relatively high level of interest in genetic information, including secondary findings, among the Korean population. Disclosure preferences were higher for treatable conditions (79.5% vs. 70.2%, *p* < 0.001), disease risk (76.3% vs. 73.2%, *p* < .001) and severity (79.7% vs. 72.0%, *p *< .001). Variable concerns included knowledge of the disease risk (31.6%), privacy issues (17.1%), emotional reactions (16.9%) and reliability of laboratory findings (10.9%), while 23.5% of the participants had no concerns. Secondary findings were diagnostic in 16 variants across 29 participants from 18 families (18/387, 4.7%) in 14 genes (Table S5 in Supporting Information 2). Among these 29 participants, only one individual did not want disclosure. The most frequently identified pathogenic variants were in the *PCSK9* (4/28, 14.3%) and *BRCA2* (4/28, 14.3%) genes, followed by the *MYL3* gene (3/28, 10.7%) (Figure 2F).

In summary, GS results had a clinical impact in 150 of the 387 families (38.8%) in this study, with primary findings in 139 families (35.9%), secondary findings in 18 families (4.7%) and both primary and secondary findings in five families (1.3%) (Figure 3A). Clinical interventions were implemented in 56 families (37.3%), including disease surveillance (29/56, 51.8%), specific medications (15/56, 26.8%), solid organ transplantation (6/56, 10.7%), other surgery (2/56, 3.6%), bone marrow transplantation (3/56, 5.4%) and family planning (1/56, 1.8%) (Figure 3B; Table S6 in Supporting Information 2). For instance, major procedures included kidney transplantation for Alport syndrome and polycystic kidney disease, heart transplantation for cardiomyopathy, and aortic valve replacement for Loeys‐Dietz syndrome and familial thoracic aortic aneurysm. However, among the 248 families with nondiagnostic or negative GS results, clinical management largely remained unchanged, except in two cases involving surgery for congenital heart disease and scoliosis correction.

**FIGURE 3:**
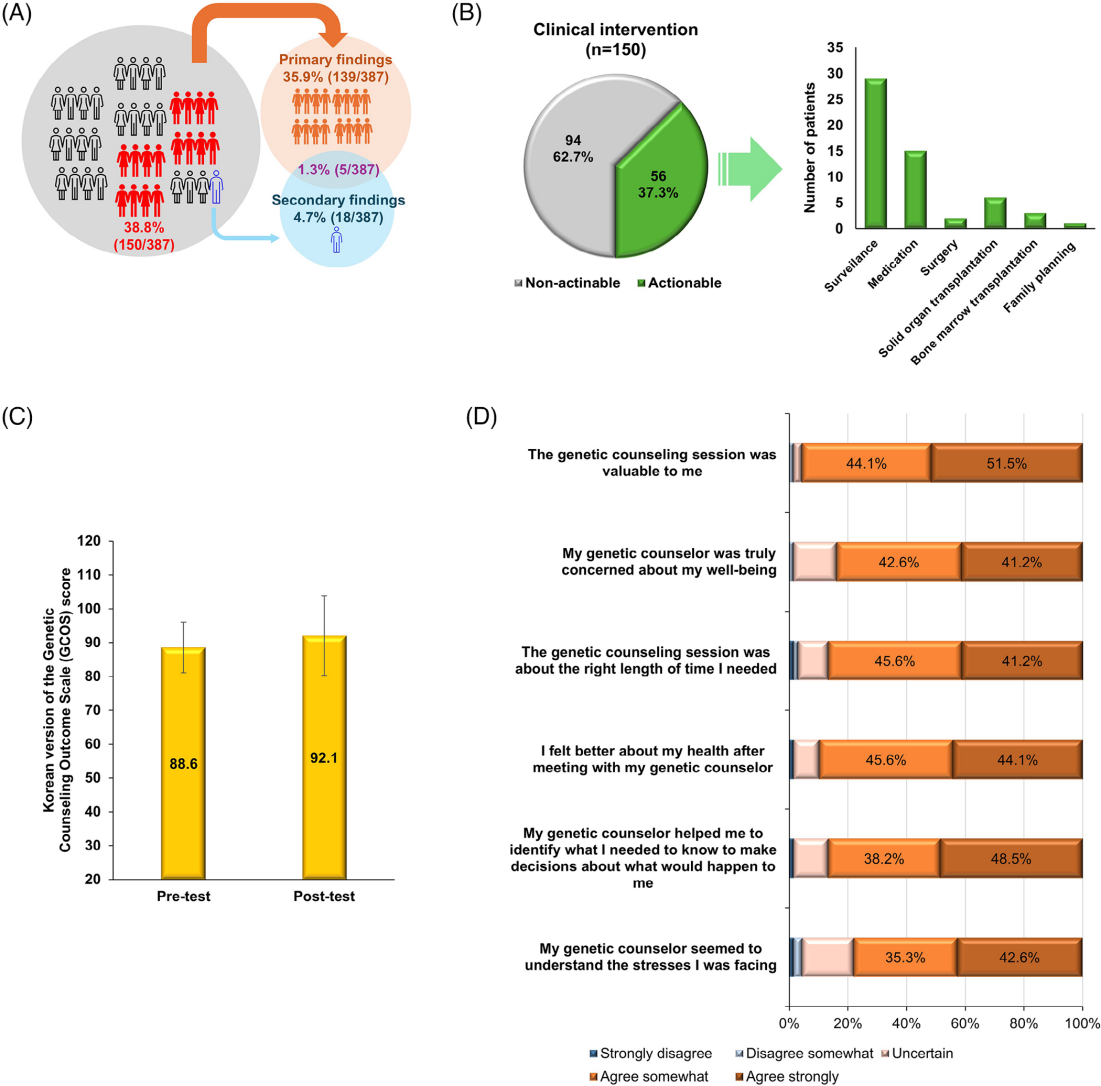
Real‐world diagnosis using genome sequencing (GS) for rare diseases and its clinical implications and genetic counselling. (A) Genetic diagnoses, including primary and secondary findings, for patients and their families in real‐world settings through GS. (B) Clinical interventions for patients and their families based on GS results. (C) Korean Genetic Counselling Outcome Scale (K‐GCOS) scores before and after GS. (D) Genetic Counselling Satisfaction Scale (GCSS) items assessing satisfaction with genetic counselling.

Eighty‐six participants received in‐depth post‐test genetic counselling from a genetic counsellor, and 68 completed all questionnaires (response rate: 79.1%; Table S7 in Supporting Information 2). Empowerment scores, measured using the Korean Genetic Counselling Outcome Scale,^7^ significantly increased after counselling, from 88.6 ± 7.5 pre‐test to 92.1 ± 11.8 post‐test (*p* < .007), reflecting a medium effect size (*d* = 0.36) (Figure 3C). Overall satisfaction with genetic counselling, assessed by the Genetic Counselling Satisfaction Scale,^8^ was high. Specifically, 51.5% of the study subjects found the service valuable, 48.5% felt the counsellor aided decision‐making, and 44.1% indicated an improved disease understanding. Perceptions of the counsellor's understanding of stress varied, with 42.6% strongly agreeing (Figure 3D).

This study had several limitations of note. First, the sample size was relatively small compared to other GS studies. Given the inherent nature of RDs, selection bias and heterogeneity, including variations in age, symptoms and family history, are likely present among the patients. Our study population consisted primarily of children (45.2%), whose phenotypic presentations may be incomplete for certain conditions, such as tumours, endocrine disorders and rheumatologic diseases. This could affect the diagnostic yield for RDs and potentially lead to underestimation of adult‐onset diseases.^9^ In addition, the findings may have limited generalizability to the broader population, particularly for adult‐onset diseases influenced by complex genetic and environmental factors. Lastly, this study did not include a cost‐effectiveness analysis or evaluate the economic outcomes of GS for RDs, which remains a significant issue due to the high costs associated with GS. Future studies should incorporate larger cohorts with comprehensive phenotypic data and well‐defined variables.

In conclusion, we have here demonstrated the beneficial outcomes of GS for RD diagnoses and management, achieved through a team‐based process that includes pre‐test surveys, systematic technical analysis, clinical assessment/confirmation and post‐test genetic counseling. Notably, our study underscores the importance of integrating professional genetic counseling into the GS process, highlighting its value in interpreting primary and secondary findings and enhancing patient‐centered care in real‐world clinical settings.

## AUTHOR CONTRIBUTIONS

**Concept and design**: Mi‐Hyun Park; Beom Hee Lee; Hyun‐Young Park. **Drafting of the manuscript**: Soojin Hwang; Go Hun Seo; In Hee Choi. **Acquisition, analysis or interpretation of the data**: Soojin Hwang; Go Hun Seo; In Hee Choi; Seung‐Woo Ryue; Ji Young Oh; Yoo‐Mi Kim; Baik‐Lin Eun; Jung Hye Byeon; Eugu Kang; Myungshin Kim; Hoon Seok Kim; Soyoung Lee; Han Wool Kim; Rin Khang; Jihye Kim; Dongseok Moon; Seokhui Jang; E. Lee; Yongjun Song; Kyoung Bo Kim; Jun Hong Park; Seo Yeon Yang; Yoo Kyoung Choi; Su Min Ji; Oc‐Hee Kim; Dohyung Kim. **Critical review of the manuscript**: Gu‐Hwan Kim; Mi‐Hyun Park; Beom Hee Lee; Hyun‐Young Park. **Statistical analysis**: Soojin Hwang; Go Hun Seo; In Hee Choi; Seung‐Woo Ryue.

## CONFLICT OF INTEREST STATEMENT

The authors declare no conflict of interest.

## FUNDING INFORMATION

This work was supported by the Research Program funded by the Korea Disease Control and Prevention Agency (grant number: 2023‐ER0705‐00) and by 3billion Inc. (Seoul, South Korea).

## ETHICS STATEMENT

This study was conducted with prior informed consent from patients or their legal guardians following thorough genetic counselling. It received approval from the Institutional Review Boards (IRBs) of all participating hospitals, including Asan Medical Center (IRB No. 2023‐0921), Severance Children's Hospital (IRB No. 4‐2023‐1062), Chungnam National University Sejong Hospital (IRB No. CNUSH2023‐07‐022), Korea University Guro Hospital (IRB No. K2023‐1969‐002), Korea University Ansan Hospital (IRB No. K2023‐1633‐001), Korea University Anam Hospital (IRB No. K2023‐2018‐002), The Catholic University of Korea Seoul St. Mary's Hospital (IRB No. KC23TNDI0736) and Hallym University Sacred Heart Hospital (IRB No. HALLYM 2023‐07‐016‐001). The research adhered to Good Clinical Practice and the principles of the Declaration of Helsinki.

## Supporting information



Supporting Information

Supporting Information

## Data Availability

The data generated or analysed in this study are available within this published article and its Supporting Information.

## References

[1] Marwaha S , Knowles JW , Ashley EA . A guide for the diagnosis of rare and undiagnosed disease: beyond the exome. Genome Med. 2022;14(1):23. doi:10.1186/s13073-022-01026-w 35220969 PMC8883622

[2] Kruse J , Mueller R , Aghdassi AA , Lerch MM , Salloch S . Genetic testing for rare diseases: a systematic review of ethical aspects. Front Genet. 2021;12:701988. doi:10.3389/fgene.2021.701988 35154238 PMC8826556

[3] Chung CCY , Hue SPY , Ng NYT , et al. Meta‐analysis of the diagnostic and clinical utility of exome and genome sequencing in pediatric and adult patients with rare diseases across diverse populations. Genet Med. 2023;25(9):100896. doi:10.1016/j.gim.2023.100896 37191093

[4] Allen NL , Karlson EW , Malspeis S , Lu B , Seidman CE , Lehmann LS . Biobank participants' preferences for disclosure of genetic research results: perspectives from the OurGenes, OurHealth, OurCommunity project. Mayo Clin Proc. 2014;89(6):738‐746. doi:10.1016/j.mayocp.2014.03.015 24943692 PMC4148696

[5] Fernandez CV , Bouffet E , Malkin D , et al. Attitudes of parents toward the return of targeted and incidental genomic research findings in children. Genet Med. 2014;16(8):633‐640. doi:10.1038/gim.2013.201 24434691

[6] Miller DT , Lee K , Abul‐Husn NS , et al. ACMG SF v3.2 list for reporting of secondary findings in clinical exome and genome sequencing: a policy statement of the American College of Medical Genetics and Genomics (ACMG). Genet Med. 2023;25(8):100866. doi:10.1016/j.gim.2023.100866 37347242 PMC10524344

[7] Yang SCY , Kim KO , Lee BH , et al. Cross‐cultutral validation of the genetic counseling outcome scale in Korea. J Genet Couns. 2025;34(2):e1961. 10.1002/jgc4.1961 PMC11923504 39175127

[8] Tercyak KP , Johnson SB , Roberts SF , Cruz AC . Psychological response to prenatal genetic counseling and amniocentesis. Patient Educ Couns. 2001;43(1):73‐84. doi:10.1016/s0738-3991(00)00146-4 11311841

[9] Taylor JC , Martin HC , Lise S , et al. Factors influencing success of clinical genome sequencing across a broad spectrum of disorders. Nat Genet. 2015;47(7):717‐726. doi:10.1038/ng.3304 25985138 PMC4601524

