# Supplementary material for: Clinical implementation of a multidisciplinary pipeline for genome sequencing in rare diseases: A prospective, multicenter, observational cohort study
Source: Clin Transl Med. 2025 Jul 10;15(7):e70401. doi: 10.1002/ctm2.70401 (PMC12245977; doi:10.1002/ctm2.70401)
Supplement: Supplementary file 1 — Supporting Information [file CTM2-15-e70401-s001.docx]

**Supplement 1**

**Clinical Implementation of a Multidisciplinary Pipeline for Genome Sequencing in Rare Diseases: A Prospective, Multicenter, Observational, Cohort Study**

**Authors**

Soojin Hwang^1†^, Go Hun Seo^2†^, In Hee Choi^3†^, Seung-Woo Ryue^2^, Ji Young Oh^4^, Yoo-Mi Kim^5^, Baik-Lin Eun^6^, Jung Hye Byeon^6^, Eugu Kang^6^, Myungshin Kim^7^, Hoon Seok Kim^7^, Soyoung Lee^8^, Han Wool Kim^8^, Dohyung Kim^1^, Rin Khang^2^, Jihye Kim^2^, Dongseok Moon^2^, Seokhui Jang^2^, Yongjun Song^2^, Gu-Hwan Kim^9^, Kyoung Bo Kim^10^, Jun Hong Park^11^, Seo Yeon Yang^3^, Yoo Kyoung Choi^3^, Su Min Ji^3^, Oc-Hee Kim^12^, Mi-Hyun Park^12*^, Hyun-Young Park^13*^, Beom Hee Lee^1*^

^†^These authors contributed equally to this work.

^*^Correspondence

**Corresponding authors:**

**Mi-Hyun Park, Ph.D.**

Division of Genome Science, Department of Precision Medicine, Korea National Institute of Health (KNIH), 187 Osongsaengmyeong 2‑ro, Osong‑eup, Heungdeok‑gu, Cheongju, North Chungcheong Province 28159, Republic of Korea

Tel: +82-43-719-8871

E-mail: [mihyun4868@korea.kr](mailto:mihyun4868@korea.kr)

**Beom Hee Lee, M.D., Ph.D.**

Medical Genetics Center, Asan Medical Center Children’s Hospital, University of Ulsan College of Medicine, 88, Olympic-ro 43-gil, Songpa-gu, Seoul 05505, South Korea

Tel: + 82-2-3010-5950, Fax: + 82-2-3010-4704

E-mail: [bhlee@amc.seoul.kr](mailto:bhlee@amc.seoul.kr)

**Hyun-Young Park, M.D., Ph.D.**

Korea National Institute of Health (KNIH), Osong Health Technology Administration Complex, 187 Osongsaengmyeong 2-ro, Osong-eup, Heungdeok-gu, Cheongju-si, Chungcheongbuk-do 28159, Republic of Korea

Tel: +82-43-719-7700

E-mail: [hypark65@korea.kr](mailto:hypark65@korea.kr)

**Methods**

***Participants and ethics statements***

This study was conducted as a follow-up study of the Korean National Bio Big Data Pilot Project, which enrolled 14,992 participants with rare diseases (RDs) between 2020 and 2022 (https://www.kobic.re.kr/kobic/res/ngp_pilot?lang=en). Our study cohort included 900 participants. The enrollment criteria for patients with RDs in this project were as follows, with inclusion based on fulfillment of at least one of the following: (1) cases with a strong genetic family history in which no affected individuals had received a definitive diagnosis; (2) cases that remained genetically undiagnosed despite undergoing prior genetic testing, including single-gene tests, karyotyping, chromosomal microarray, multiplex ligation-dependent probe amplification (MLPA), next-generation sequencing (NGS) panels, or exome sequencing (ES); and (3) cases in which an initial diagnosis was established through prior testing, but the patient exhibited atypical clinical features or an unusual disease course. The study enrolled 901 individuals from 387 families across eight South Korean hospitals between August and November 2023. The demographic, laboratory, and radiologic findings for these subjects were reviewed. The entire genome of each participant was sequenced and analyzed with informed consent. Patients or their legal guardians had the option to be informed of medically actionable secondary findings as defined by the American College of Medical Genetics and Genomics (ACMG) guidelines (ACMG SF v3.2 list).(1) This study was approved by the Institutional Review Boards of each participating hospital and was conducted according to Good Clinical Practice and in agreement with the principles of the Helsinki Declaration.

***Genome sequencing and analysis***

Whole blood samples were collected from each proband and their family members. Genomic DNA was extracted from blood samples using the QIAamp DNA Blood Mini Kit (Qiagen, version 4.2). Genome sequencing was performed at Macrogen, Inc. using the TruSeq DNA PCR–free sample preparation kit (Illumina, San Diego, CA) on the NovaSeq 6000 system (Illumina) with 150 base pair (bp) paired-end sequencing. Sequencing data analysis was performed at 3billion Inc. Base call (BCL) sequence files were converted and demultiplexed to FASTQ files using bcl2fastq v2.20.0.422 (Illumina). Sequence reads were aligned to the Genome Reference Consortium Human Build 38 (GRCh38) and Revised Cambridge Reference Sequence (rCRS) of the mitochondrial genome using BWA-mem2 to generate BAM files. Aligned BAM files were sorted, and duplicates were marked using Samtools v.1.15.(2) Recalibration and variant calling for single-nucleotide variants (SNVs) and small insertion/deletion variants (INDELs) were performed using GATK v4.2.14.(3, 4) Mutect2 v4.1.0.0 was used for SNV and INDEL calling for the mitochondrial genome.(5) Structural variants (SVs), including copy number variants (CNVs), inversions, and translocations, were identified using MANTA v1.6.0 and 3bCNV, a tool developed by 3billion based on the depth-of-coverage (DOC) information.(6) The number of chromosomes was predicted based on the DOC information for the detection of aneuploidy. Deletions of the SMN1 gene were called using the SMA finder.(7) Short tandem repeat expansions (STREs) were limited to the calling of 45 genes (AFF2, AR, ARX, ATN1, ATXN10, ATXN1, ATXN2, ATXN3, ATXN7, ATXN8OS, BEAN1, C9ORF72, CACNA1A, CNBP, COMP, DAB1, DIP2B, DMPK, FGF14, FMR1, FOXL2, FXN, GIPC1, GLS, HOXD13, HTT, JPH3, LRP12, MARCHF6, NOP56, NOTCH2NLC, NUTM2B-AS1, PABPN1, PHOX2B, PPP2R2B, PRDM12, RAPGEF2, RFC1, RILPL1, SAMD12, STARD7, TBP, TCF4, XYLT1, and ZIC2) from ExpansionHunter v5.0.0 using RepeatCatalogs-v1.0.022.(8) Mobile element insertions (MEIs) were called using Mobile Element Locator Tool (MELT) v2.2.2.(9) AutoMap v1.2 was used to detect regions of homozygosity (ROH).(10) The mean DOC was 34.7× per genome with a minimum of 96.8% coverage at 20× DOC.

***Overall diagnostic process from interpretation to report***

Figure 1 outlines the entire process used in the study from variant interpretation to reporting. Variants were annotated, filtered, and prioritized using the EVIDENCE automatic interpretation pipeline developed by 3billion, Inc. Although not open-source, it can be accessed by researchers and clinicians through a formal request or collaboration process. The pipeline includes a daily updated database module, a customized variant classification module, and a symptom similarity scoring module. Additionally, a demo version of 3billion's GEBRA platform is available for users to explore via the 3billion website ([https://3billion.io](https://3billion.io/)).(11) Variants were annotated with a variant effect predictor using the latest databases.(12) The selection of representative transcripts was based on the Matched Annotation from NCBI and EMBL-EBI or on canonical transcript information in the RefSeq database (and the Ensembl database when not available in the former).(13) In cases without canonical transcript annotations, transcripts with the most severe predicted consequence were selected. Previously reported variants, at least in the literature, or reported as pathogenic (P) or likely pathogenic (LP) in ClinVar (<https://www.ncbi.nlm.nih.gov/clinvar/>) were excluded from the filtering process. SNVs/INDELs with an allele frequency (AF) > 5% in gnomAD v3.0 (<https://gnomad.broadinstitute.org/>), CNVs with an AF > 1% in gnomAD SVs v2.1 (https://gnomad.broadinstitute.org/) and in the Database of Genomic Variants (DGV) gold standard (<https://dgv.tcag.ca/>), and SVs with an AF> 1% in 3billion, Inc. database were removed initially. The remaining SNV/INDELs and CNVs in coding and non-coding regions, including novel variants, not previously described in the literature or contained in the aforementioned databases, were then classified as P, LP, variant of uncertain significance (VUS), likely benign (LB), or benign (B) in accordance with the customized ACMG and the American Molecular Pathology guidelines.(14-16) Breakpoints for inversions and translocations were within known disease genes, or large inversions were considered as potentially disease-causing. For STREs, the repeats were considered pathogenic if the repeat number was equal to or greater than the normal repeat number range reported in the literature or the iSTRipy database.(17) MEIs were considered to be potentially disease-causing if the insertion occurred within a coding exon or a known non-coding region known as disease-causing.(18) The extent to which each variant matched the patient's symptoms with those of a known disease was assessed by calculating the symptom similarity via a modification of the original method using depth of Human Phenotype Ontology terms instead of the information content of terms described in those prior studies.(11, 19) A positive report consisted of one or more disease-causing variants determined as follows: for an autosomal dominant disease or an X-linked disease, one heterozygous or hemizygous P/LP variant in a known disease gene that would fit the phenotype, and for an autosomal recessive disease, one homozygous P/LP variant or two P/LP compound heterozygous variants in a relevant gene. An inconclusive report included cases with one heterozygous or hemizygous VUS in a known autosomal dominant or X-linked disease gene, or cases having potential compound heterozygous variants with one P/LP variant and one VUS, a homozygous VUS variant, or only one P/LP variant in a known autosomal recessive disease gene. If P/LP variants were found in a gene that had not been previously associated with a disease defined in the Online Mendelian Inheritance in Man but reported in the literature, they were classified as inconclusive. Negative reports were issued when no clinically significant variants were found. Medical geneticists manually reviewed and validated reportable variants, which were then examined using the Integrative Genomics Viewer (IGV, version 2.9.4).(2) Clinicians reviewed these findings and provided feedback, leading to the final diagnostic report for each proband.

***Genetic counseling and follow-up patient care***

The study participants included patients with genetic disorders, unaffected individuals with affected relatives, and parents of affected children, all aged 18 years or older. Individuals with cognitive impairments were excluded. Genetic counseling sessions covered the interpretation of genomic results, diagnosis and treatment of conditions, disease management, psychosocial support, and guidance on family testing or prenatal diagnostics. Genetic counselors collected patient medical histories, created pedigrees, and communicated medical and genetic information from medical geneticists in understandable terms. Each session, lasting 60 minutes per case, was provided free of charge. All participants underwent pre-test counseling conducted by either a physician or a genetic counselor prior to GS. After receiving the final diagnostic report, medical geneticists and certified genetic counselors provided detailed genetic counseling to participants regarding both primary and secondary genomic findings. Empowerment was assessed using the validated Korean version of the Genetic Counseling Outcome Scale (K-GCOS), which includes 20 of the original 24 questions and measures cognitive-behavioral control, uncertainty management, hope, and emotional regulation.(20) Scores range from 20 to 140 on a seven-point Likert scale, with higher scores indicating greater empowerment. Satisfaction with genetic counseling services was evaluated using the Genetic Counseling Satisfaction Scale (GCSS).(21) The GCSS comprises six items rated on a 5-point Likert scale, with higher scores indicating higher satisfaction. Participants completed both pre- and post-test K-GCOS and GCSS surveys online. To assess awareness and attitudes toward secondary findings, we adapted and supplemented tools from previous genomic studies to fit the objectives of this study.(22, 23) The Cronbach’s alpha for this study was 0.911.

**References**

1. Miller DT, Lee K, Abul-Husn NS, Amendola LM, Brothers K, Chung WK, et al. ACMG SF v3.2 list for reporting of secondary findings in clinical exome and genome sequencing: A policy statement of the American College of Medical Genetics and Genomics (ACMG). Genet Med. 2023;25(8):100866.

2. Li H, Handsaker B, Wysoker A, Fennell T, Ruan J, Homer N, et al. The Sequence Alignment/Map format and SAMtools. Bioinformatics. 2009;25(16):2078-9.

3. McKenna A, Hanna M, Banks E, Sivachenko A, Cibulskis K, Kernytsky A, et al. The Genome Analysis Toolkit: A MapReduce framework for analyzing next-generation DNA sequencing data. Genome Res. 2010;20(9):1297-303.

4. DePristo MA, Banks E, Poplin R, Garimella KV, Maguire JR, Hartl C, et al. A framework for variation discovery and genotyping using next-generation DNA sequencing data. Nat Genet. 2011;43(5):491-+.

5. Cibulskis K, Lawrence MS, Carter SL, Sivachenko A, Jaffe D, Sougnez C, et al. Sensitive detection of somatic point mutations in impure and heterogeneous cancer samples. Nat Biotechnol. 2013;31(3):213-9.

6. Chen XY, Schulz-Trieglaff O, Shaw R, Barnes B, Schlesinger F, Källberg M, et al. Manta: rapid detection of structural variants and indels for germline and cancer sequencing applications. Bioinformatics. 2016;32(8):1220-2.

7. Weisburd B, Sharma R, Pata V, Reimand T, Ganesh VS, Austin-Tse C, et al. Detecting missed diagnoses of spinal muscular atrophy in genome, exome, and panel sequencing datasets. medRxiv. 2024:2024.02.11.24302646.

8. Dolzhenko E, Deshpande V, Schlesinger F, Krusche P, Petrovski R, Chen S, et al. ExpansionHunter: a sequence-graph-based tool to analyze variation in short tandem repeat regions. Bioinformatics. 2019;35(22):4754-6.

9. Gardner EJ, Lam VK, Harris DN, Chuang NT, Scott EC, Pittard WS, et al. The Mobile Element Locator Tool (MELT): population-scale mobile element discovery and biology. Genome Res. 2017;27(11):1916-29.

10. Quinodoz M, Peter VG, Bedoni N, Bertrand BR, Cisarova K, Salmaninejad A, et al. AutoMap is a high performance homozygosity mapping tool using next-generation sequencing data. Nat Commun. 2021;12(1).

11. Seo GH, Kim T, Choi IH, Park JY, Lee J, Kim S, et al. Diagnostic yield and clinical utility of whole exome sequencing using an automated variant prioritization system,EVIDENCE. Clin Genet. 2020;98(6):562-70.

12. McLaren W, Gil L, Hunt SE, Riat HS, Ritchie GRS, Thormann A, et al. The Ensembl Variant Effect Predictor. Genome Biol. 2016;17.

13. Wright CF, FitzPatrick DR, Ware JS, Rehm HL, Firth HV. Importance of adopting standardized MANE transcripts in clinical reporting. Genet Med. 2023;25(2).

14. Richards S, Aziz N, Bale S, Bick D, Das S, Gastier-Foster J, et al. Standards and guidelines for the interpretation of sequence variants: a joint consensus recommendation of the American College of Medical Genetics and Genomics and the Association for Molecular Pathology. Genet Med. 2015;17(5):405-24.

15. Riggs ER, Andersen EF, Cherry AM, Kantarci S, Kearney H, Patel A, et al. Technical standards for the interpretation and reporting of constitutional copy-number variants: a joint consensus recommendation of the American College of Medical Genetics and Genomics (ACMG) and the Clinical Genome Resource (ClinGen). Genet Med. 2020;22(2):245-57.

16. McCormick EM, Lott MT, Dulik MC, Shen LS, Attimonelli M, Vitale O, et al. Specifications of the ACMG/AMP standards and guidelines for mitochondrial DNA variant interpretation. Hum Mutat. 2020;41(12):2028-57.

17. Halman A, Dolzhenko E, Oshlack A. STRipy: A graphical application for enhanced genotyping of pathogenic short tandem repeats in sequencing data. Hum Mutat. 2022;43(7):859-68.

18. Jaganathan K, Panagiotopoulou SK, McRae JF, Darbandi SF, Knowles D, Li YI, et al. Predicting Splicing from Primary Sequence with Deep Learning. Cell. 2019;176(3):535-+.

19. Köhler S, Schulz MH, Krawitz P, Bauer S, Dölken S, Ott CE, et al. Clinical Diagnostics in Human Genetics with Semantic Similarity Searches in Ontologies. Am J Hum Genet. 2009;85(4):457-64.

20. Yang S CY, Kim KO, Lee BH, Kong SY, Mcallister M, et al. Cross-cultutral validation of the genetic counseling outcome scale in Korea. J Genet Couns. 2024.

21. Tercyak KP, Johnson SB, Roberts SF, Cruz AC. Psychological response to prenatal genetic counseling and amniocentesis. Patient Educ Couns. 2001;43(1):73-84.

22. Allen NL, Karlson EW, Malspeis S, Lu B, Seidman CE, Lehmann LS. Biobank participants' preferences for disclosure of genetic research results: perspectives from the OurGenes, OurHealth, OurCommunity project. Mayo Clin Proc. 2014;89(6):738-46.

23. Fernandez CV, Bouffet E, Malkin D, Jabado N, O'Connell C, Avard D, et al. Attitudes of parents toward the return of targeted and incidental genomic research findings in children. Genet Med. 2014;16(8):633-40.
